# Contralateral Recurrent Laryngeal Nerve Palsy in Primary Anterior Cervical Corpectomy and Fusion: A Case Report

**DOI:** 10.7759/cureus.91867

**Published:** 2025-09-08

**Authors:** Natalie M Kistler, Rafael Guizar III, William Karakash, Henry Avetisian, Matthew Gallo, Ram K Alluri

**Affiliations:** 1 Department of Orthopaedic Surgery, University of Southern California Keck School of Medicine, Los Angeles, USA; 2 Department of Orthopaedic Surgery, University at Buffalo Jacobs School of Medicine and Biomedical Sciences, Buffalo, USA

**Keywords:** anterior cervical corpectomy and fusion, cervical spine, preoperative evaluation, recurrent laryngeal nerve palsy, vocal cord paralysis

## Abstract

Recurrent laryngeal nerve palsy (RLNP) is a well-known complication of anterior cervical spine surgery, particularly in revision procedures, and can result in hoarseness, dysphagia, or complete vocal cord paralysis if severe. This complication typically presents on the side of the surgical approach. We present a case of a 76-year-old male with a rare contralateral RLNP following primary anterior cervical corpectomy and fusion (ACCF). This RLNP was undiagnosed before presentation at our clinic and was identified through preoperative otolaryngology consultation and flexible laryngoscopic examination. Therefore, we decided to perform a revision ACCF with hardware removal at C3-C6 using a left-sided approach to mitigate the risk of bilateral vocal cord paralysis. The case highlights the significance of preoperative laryngoscopy to diagnose and localize a potential RLNP before revision surgery, thereby reducing the risk of potentially life-threatening consequences of bilateral RLNP. These findings challenge the existing assumption that RLNP following primary ACCF occurs only on the side of the surgical approach, emphasizing the need for heightened vigilance in surgical planning and decision-making for revision anterior cervical spine procedures.

## Introduction

Anterior cervical spine surgery, including anterior cervical discectomy and fusion (ACDF) and anterior cervical corpectomy and fusion (ACCF), is considered the gold standard in surgical management of degenerative cervical spine diseases [1]. A relatively uncommon complication of anterior cervical spine surgery is recurrent laryngeal nerve palsy (RLNP), which typically occurs on the side of the surgical approach, with a reported incidence ranging from 0.9% to 8.3% [2-5]. RLNP can result directly from surgical injury or indirectly from compression or tension related to retractor placement, endotracheal cuff inflation, or intubation [3,6]. While unilateral RLN injury can be a subclinical finding in asymptomatic patients, clinical manifestations of RLNP include hoarseness, cough, or dysphagia. In cases of bilateral RLNP, this condition can progress to complete vocal cord paralysis and respiratory failure, requiring rapid airway management and tracheostomy [3,6]. Several risk factors for postoperative RLNP have been identified, including revision or multilevel procedures, increased endotracheal tube cuff pressure, prolonged surgery duration, and a right-sided approach [2-5].

This report presents a rare case of contralateral RLNP following primary ACCF, which, to the authors’ knowledge, is a complication that has not been previously reported in the literature. The case highlights the critical importance of preoperative laryngoscopy to diagnose and localize a potential RLNP before revision surgery, thereby limiting the risk of potentially life-threatening consequences of bilateral RLNP. These findings challenge the existing assumption that RLNP following primary ACCF occurs on the side of the surgical approach, emphasizing the need for heightened vigilance in surgical planning and decision-making for revision procedures. 

## Case presentation

A 76-year-old male with a past medical history of hypertension, hyperlipidemia, metastatic prostate cancer, and tonsillar cancer treated with the combination of radiation, tonsillectomy, and a salvage left neck dissection over 20 years ago presented to the spine clinic with six months of worsening neck pain and dysphagia. In 2021, he had undergone a right-sided C3-C6 ACCF at an outside facility for a reported insufficiency fracture. Immediately following the procedure, he developed worsening dysphonia and dysphagia, which never fully resolved. Before surgery, he had no dysphonia or dysphagia.

**Figure 1:**
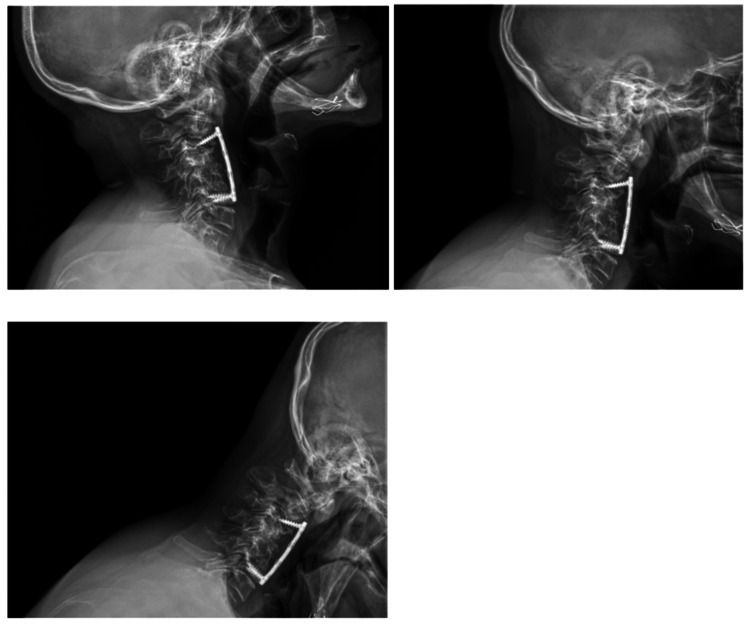
Preoperative X-ray of cervical spine

**Figure 2:**
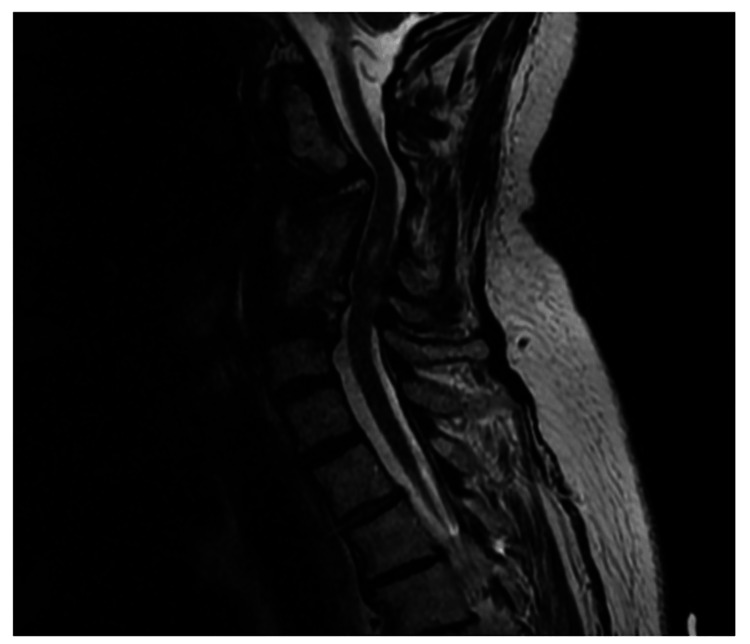
Preoperative T2 MRI showing C3-C6 cord compression with focal myelomalacia

On physical examination, the patient exhibited axial neck pain exacerbated by cervical flexion and extension. The neurological examination was unremarkable with intact sensation and strength and negative Spurling’s and Hoffman’s signs. Radiographs and CT imaging demonstrated screw cut-out proximally and focal kyphosis at the C3 level (Figure 1). T2 MRI imaging confirmed moderate-to-severe cord compression from C3 to C6 with focal myelomalacia (Figure 2).

Given the patient’s history of immediate postoperative dysphonia, RLNP was suspected. Otorhinolaryngology consultation was obtained, and flexible laryngoscopy revealed left true vocal cord paralysis with significant secretions in the pyriform sinus.

Given the imaging findings of severe cord compression and the patient’s worsening hand numbness, the decision was made to perform a revision ACCF with hardware removal at C3-C6. To mitigate the risk of bilateral vocal cord paralysis, a left-sided approach was chosen for the revision ACCF, despite the initial right-sided approach during the index ACCF. A two-stage procedure was planned to provide increased stability through an anterior and posterior construct, compensating for the patient’s poor bone quality. The first stage involved a left-sided anterior approach with revision C3-C6 corpectomy, expandable cage placement, and C2-C7 anterior plating. The second stage consisted of a C3-C6 laminectomy and C2-T2 posterior fusion. The primary goals of the surgery were to improve cervical alignment by revising the failed hardware and providing a stable construct.

**Figure 3:**
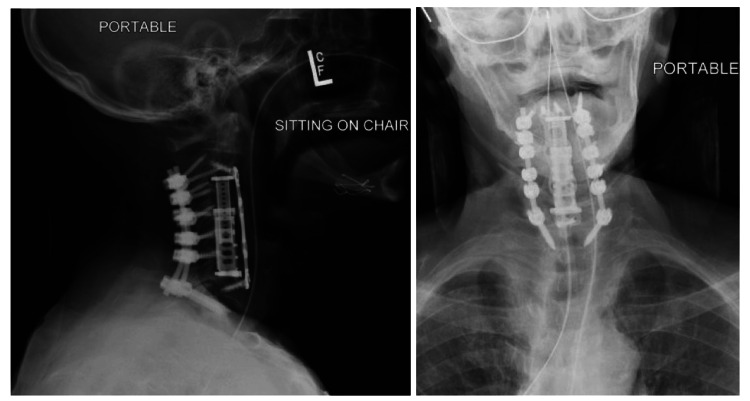
Postoperative X-rays indicating correct hardware placement

Despite the increased risks posed by the patient’s comorbidities, poor bone quality, and immunocompromised state, the patient tolerated the procedure well. Postoperative imaging confirmed intact hardware and maintained spine alignment (Figure 3). The team was unable to appropriately assess the patient's recovery of symptoms, such as dysphagia and dysphonia, because the patient left against medical advice shortly following the procedure.

## Discussion

Symptomatic RLNP is a well-known complication of anterior cervical spine surgery [2]. Various mechanisms of injury have been proposed, including increased endotracheal tube pressure on the submucosa, retraction-induced neuropraxia, airway trauma during intubation, or nerve division or ligation [3,6-8]. The risk is higher in revision surgery due to scar tissue from previous surgeries, which can make the nerves more susceptible to damage [7].

In our patient’s case, his distant history of tonsillar cancer, radiation, and radical left neck dissection likely contributed to the contralateral RLNP by increased scar tissue and fibrosis, making the nerve more vulnerable during the primary ACCF [9]. Radiation therapy is a documented risk factor for RLNP, with Carpenter et al. reporting a unilateral RLNP following radiotherapy to the thorax’s apex and Woodacre et al. identifying an asymptomatic RLNP following radiotherapy for apical lung cancer [10,11]. Wound contraction during scar formation following primary dissection or radiation therapy can alter the anatomical position of the nerve and increase adhesion to surrounding structures, heightening the risk of unintentional injury [5]. Additionally, fibrosis can impair the vascular blood supply to nerves, potentially disrupting their function and further increasing their susceptibility to damage [12]. In our patient’s case, dysphonia presented following primary ACDF, demonstrating that the RLNP was likely attributable to the primary ACDF rather than pre-existing from the patient’s radiation therapy and neck dissection. However, it is also possible that the patient had a subclinical RLNP following cancer treatment that worsened after the primary ACDF.

Although RLNP can present with immediate postoperative symptoms such as hoarseness, cough, and dysphagia, it may also manifest in a delayed fashion or remain asymptomatic. Studies have shown that up to one-third of affected patients may have asymptomatic vocal fold motion impairment, and some patients who are initially symptomatic may become asymptomatic over time [13-17]. This variability in presentation complicates the decision-making process for revision procedures.

Patients undergoing anterior cervical spine surgery, particularly revision procedures, face a significant risk of bilateral RLNP, especially if they have an asymptomatic or misdiagnosed unilateral RLNP. This potentially life-threatening complication can lead to airway obstruction, aphonia, and respiratory distress, and may require urgent or emergent interventions such as tracheostomy, which could dramatically impact patients' long-term quality of life [8,10,15]. Given the prevalence of RLNP following anterior cervical spine surgery, preoperative laryngoscopy has been recommended for symptomatic patients undergoing revision surgery [15]. This screening method can effectively distinguish RLNP from other causes of dysphonia, evaluate the posterior cricoarytenoid muscle to predict potential RLNP, and inform clinical decision-making regarding the side of surgical approach [13,16,17]. Implementation of a mandatory preoperative laryngoscopic examination in a cohort of revision ACDF candidates identified asymptomatic RLNPs in 17.3% of patients and positively informed decision-making regarding the side of revision approach [15]. Despite its utility, the implementation of preoperative laryngoscopic examinations in the setting of revision anterior cervical spine surgery remains limited. 

The critical importance of preoperative laryngoscopy in symptomatic patients undergoing revision anterior cervical spine surgery is illustrated by our patient's case. Despite reporting hoarseness and dysphagia since his initial ACCF in 2021, the patient's potential nerve injury went undiagnosed until his presentation at our institution. Our high clinical suspicion for RLNP prompted a preoperative laryngoscopy, revealing a surprising and crucial finding: a contralateral RLNP, likely stemming from the primary procedure. 

While ipsilateral RLNP is a well-documented complication of anterior cervical spine surgery, contralateral RLNP following a primary anterior cervical spine surgery remains largely unreported in the literature. To date, only two remotely similar cases have been described: Wu et al. reported a case of postoperative contralateral RLNP in revision ACDF without preoperative vocal cord paresis, and Woodacre et al. documented bilateral vocal cord paralysis following a primary right-sided ACDF, attributing an underlying left RLNP to the patient's history of radiotherapy for left apical lung cancer [8,10].

Our case represents an unprecedented scenario that challenges existing assumptions about RLNP in anterior cervical spine surgery. Without the preoperative laryngoscopy, the standard assumption would have been a right-sided RLNP, corresponding to the side of the initial surgical approach. Such an assumption, though logical based on current literature, would have led to the decision to use a right-sided approach for the revision surgery, placing the patient at undue risk for bilateral vocal cord paralysis. The laryngoscopy's unexpected finding fundamentally altered the surgical strategy, directing us toward a left-sided approach and averting a potentially life-threatening outcome. 

## Conclusions

This case underscores the important role of preoperative laryngoscopy in revision anterior cervical spine surgeries, especially in symptomatic patients or those with a history of risk factors such as previous neck or spine surgery. Preoperative evaluation can help diagnose and localize a potential RLNP before revision surgery, thereby reducing the risk of potentially life-threatening consequences of bilateral RLNP. Moreover, this case emphasizes the need for heightened awareness of the possibility of contralateral RLNP, even in primary ACCF cases, which challenges the existing assumption that RLNP following primary ACCF occurs only on the side of the surgical approach. This case calls for increased interdisciplinary collaboration between spine surgeons and otolaryngologists to optimize preoperative care. Future studies are needed to better define patient-selection criteria and clinical guidelines for preoperative laryngeal evaluation in anterior cervical spine surgeries.
